# Unstandardized Treatment of Electroencephalographic Status Epilepticus Does Not Improve Outcome of Comatose Patients after Cardiac Arrest

**DOI:** 10.3389/fneur.2014.00039

**Published:** 2014-03-31

**Authors:** Jeannette Hofmeijer, Marleen C. Tjepkema-Cloostermans, Michiel J. Blans, Albertus Beishuizen, Michel J. A. M. van Putten

**Affiliations:** ^1^Clinical Neurophysiology, MIRA Institute for Biomedical Technology and Technical Medicine, University of Twente, Enschede, Netherlands; ^2^Department of Neurology, Rijnstate Hospital, Arnhem, Netherlands; ^3^Department of Clinical Neurophysiology, Medisch Spectrum Twente, Enschede, Netherlands; ^4^Department of Intensive Care, Rijnstate Hospital, Arnhem, Netherlands; ^5^Department of Intensive Care, Medisch Spectrum Twente, Enschede, Netherlands

**Keywords:** continuous EEG, cardiac arrest, post-anoxic coma, status epilepticus, epileptic seizures, anti-epileptic drugs, prognosis

## Abstract

**Objective:** Electroencephalographic status epilepticus occurs in 9–35% of comatose patients after cardiac arrest. Mortality is 90–100%. It is unclear whether (some) seizure patterns represent a condition in which anti-epileptic treatment may improve outcome, or severe ischemic damage, in which treatment is futile. We explored current treatment practice and its effect on patients’ outcome.

**Methods:** We retrospectively identified patients that were treated with anti-epileptic drugs from our prospective cohort study on the value of continuous electroencephalography (EEG) in comatose patients after cardiac arrest. Outcome at 6 months was dichotomized between “good” [cerebral performance category (CPC) 1 or 2] and “poor” (CPC 3, 4, or 5). EEG analyses were done at 24 h after cardiac arrest and during anti-epileptic treatment. Unequivocal seizures and generalized periodic discharges during more than 30 min were classified as status epilepticus.

**Results:** Thirty-one (22%) out of 139 patients were treated with anti-epileptic drugs (phenytoin, levetiracetam, valproate, clonazepam, propofol, midazolam), of whom 24 had status epilepticus. Dosages were moderate, barbiturates were not used, medication induced burst-suppression not achieved, and treatment improved electroencephalographic status epilepticus patterns temporarily (<6 h). Twenty-three patients treated for status epilepticus (96%) died. In patients with status epilepticus at 24 h, there was no difference in outcome between those treated with and without anti-epileptic drugs.

**Conclusion:** In comatose patients after cardiac arrest complicated by electroencephalographic status epilepticus, current practice includes unstandardized, moderate treatment with anti-epileptic drugs. Although widely used, this does probably not improve patients’ outcome. A randomized controlled trial to estimate the effect of standardized, aggressive treatment, directed at complete suppression of epileptiform activity during at least 24 h, is needed and in preparation.

## Introduction

Of comatose patients after cardiac arrest, admitted on the intensive care unit, 40–66% never regains consciousness as a result of diffuse post-anoxic encephalopathy (1–3). In these patients, a broad spectrum of electroencephalography (EEG) changes can be observed (4). Electroencephalographic seizures or status epilepticus is described in 9–35% (4–7) and is associated with poor outcome: case fatality was 90–100% in prospective case series, despite treatment with anti-epileptic drugs (2, 6, 8–13).

The diagnosis of seizures and status epilepticus on the EEG of comatose patients after cardiac arrest is controversial (14, 15). It may consist of unequivocal seizures: generalized spike–wave discharges at 3/s or faster or clearly evolving discharges of any type at 4/s or faster, either generalized or focal. However, some experts also consider other rhythmic or periodic patterns, such as generalized or lateralized periodic discharges or rhythmic delta activity, as seizure activity (16).

It is unclear whether (some) electroencephalographic seizure patterns in patients with post-anoxic encephalopathy represent a condition, which can be treated with anti-epileptic drugs to improve patients’ outcome, or rather reflect severe ischemic damage, in which treatment is futile (17). Case series have suggested that in patients with electroencephalographic status epilepticus, preserved brainstem reactions and EEG reactivity are associated with a favorable outcome (6). However, it is unclear whether treatment with anti-epileptic drugs reduces the risk of a poor outcome in these patients. In the only prospective non-randomized intervention study, aggressive treatment up to pentobarbital induced burst-suppression resulted in a favorable outcome of 6% of patients with clinically overt or electroencephalographic status epilepticus (unpublished data). This proportion is approximately the same as reported in observational studies, irrespective of treatment (6, 10–13). Despite this lack of evidence, most neurologists treat electroencephalographic seizures and status epilepticus in comatose patients after cardiac arrest with anti-epileptic drugs. Increased detection with continuous EEG monitoring has led to increased prescription (18, 19). However, only one-third treats these patients equally aggressive to those with clinically overt status epilepticus (18, 20).

We evaluated current treatment practice, including its effects on the EEG and patients’ outcome, of seizures and electroencephalographic status epilepticus on continuous EEG in comatose patients after cardiac arrest. We used data from our prospective cohort study on the prognostic value of continuous EEG monitoring on the intensive care unit.

## Materials and Methods

### Patients

We retrospectively identified patients who were treated with anti-epileptic drugs (phenytoin, levetiracetam, valproate, clonazepam, or barbiturates) for electroencephalographic seizures or status epilepticus from our prospectively collected cohort of comatose patients after cardiac arrest, treated with mild therapeutic hypothermia, between June 1, 2010 and March 31, 2013. These patients were included in a prospective cohort study on the predictive value of continuous EEG on outcome in two teaching hospitals in the Netherlands. Design, eligibility criteria, and main outcomes of the first 60 patients that were included in this study have been published previously (4). In brief, since June 1, 2010, consecutive adult comatose patients after cardiac arrest, treated with mild therapeutic hypothermia, were included within 12 h after the arrest to undergo continuous EEG monitoring on the intensive care unit. Monitoring continued until patients regained consciousness, died, or up to 5 days. The institutional review board (Medisch Ethische Toetsingscommissie Twente) waived the need for informed consent for EEG monitoring and follow-up by telephone.

### Treatment

Comatose patients after cardiac arrest were treated according to current standard therapy, as described previously (4). In short, mild therapeutic hypothermia was induced as soon as possible after the arrest and maintained for 24 h by intravenously administered cold saline and cooling pads. Propofol, midazolam, or a combination of these was used for sedation to a level of −4 or −5 at the Richmond Agitation Sedation Scale and discontinued after normothermia had been reached, if possible. Fentanyl, remifentanil, morphine, or a non-depolarizing muscle relaxant was used against shivering.

Treatment of epileptiform discharges was not included in the study protocol. In both hospitals, decisions with regard to treatment of electroencephalographic epileptic phenomena were made by the treating intensive care physician in consultation with a neurologist/clinical neurophysiologist. Although both hospitals adhere to national guidelines for the treatment of epileptic status in general, there are no guidelines with respect to these EEG phenomena in patients with post-anoxic encephalopathy on the ICU. This indicates that both the decision to start anti-epileptic treatment, and the choice of the drugs, and the intensity of treatment were decided by the treating physician. Propofol or midazolam was identified as anti-epileptic treatment, if dosages were increased simultaneously with the initiation of treatment with anti-epileptic drugs.

### EEG recordings

For all recordings, electrodes were applied according to the international 10/20 system, using 19 channels. Electrode impedances were kept below 5 kΩ. Sampling frequency was set to 256 Hz. A Neurocenter EEG system (Clinical Science Systems, Netherlands) or a Nihon Kohden system (VCM Medical, Netherlands) was used. Data were stored to disk for off-line analysis.

### Outcome assessment

Outcome assessment was done at 3 and 6 months after cardiac arrest by telephone by a single investigator (Marleen C. Tjepkema-Cloostermans), who was blinded for treatment with anti-epileptic drugs. The primary outcome measure of the study was the best score on the cerebral performance category (CPC) within 6 months, dichotomized between “good” (CPC 1 or 2) and “poor” (CPC 3, 4, or 5). Secondary outcome measures included mortality.

### EEG analysis

Patients underwent continuous EEG monitoring, starting within 12 h after cardiac arrest, continuing until they regained consciousness, died, or up to 5 days. Real time EEG analysis was done intermittently by the consulting neurologist at the bedside, two to three times a day. Treating physicians decided on treatment with anti-epileptic drugs based on this bedside EEG analysis. However, at that time, the EEG was not used for decisions with regard to discontinuation of intensive care treatment.

Standardized, *post hoc*, off-line EEG analyses were performed at 24 h after cardiac arrest in automatically selected 5 min epochs. Additionally, off-line EEG analyses were done at the initiation of treatment with each anti-epileptic drug, with access to the full EEG recordings. Epochs at 24 h after cardiac arrest were analyzed independently by two investigators (Marleen C. Tjepkema-Cloostermans, Michel J. A. M. van Putten). Selection of the 5 min epochs was done by the computer and only based on absence of artifacts. Each epoch was categorized as iso-electric, low voltage, burst-suppression, diffuse slowing, normal, or epileptiform discharges. Epileptiform discharges included unequivocal, evolving seizures, and generalized periodic discharges (GPDs). The investigators were blinded for the patient’s clinical condition during the registration, the recording time of the epoch, and the patient’s outcome. In case of disagreement, the final classification was decided by consensus. All EEGs of patients who had been treated with anti-epileptic drugs were subsequently reviewed to classify the EEG pattern at the initiation of the treatment with each additional anti-epileptic drug and to assess its effects on EEG patterns. This was done by two observers (Jeannette Hofmeijer, Michel J. A. M. van Putten), who had access to the complete recordings, but were blinded for the patients’ outcome. Electroencephalographic status epilepticus was defined as unequivocal seizures (generalized spike–wave discharges at 3/s or faster or clearly evolving discharges of any type at 4/s or faster) or other rhythmic or periodic patterns, such as generalized or lateralized periodic discharges or rhythmic delta activity, during more than 30 min. Improvement of the EEG pattern was defined as disappearance of epileptiform discharges for at least 30 min after the start of any anti-epileptic treatment. Improvement of background pattern was not taken into account.

### Statistical analysis

The number of patients treated with the various anti-epileptic drugs, the proportion of patients in whom treatment improved EEG patterns, and the proportion of patients with a poor outcome after treatment are presented in a descriptive way for subgroups according to EEG patterns at the time of treatment initiation. Patients treated with and without anti-epileptic drugs are compared with regard to poor outcome. These comparisons are done for subgroups according to the EEG patterns observed at 24 h after cardiac arrest. Data are presented as proportions and odds ratios, including corresponding 95% confidence intervals. Comparison of baseline characteristics was done by Student’s *t*-test, Chi-square test, or Fisher’s exact test, where appropriate.

## Results

**Figure 1:**
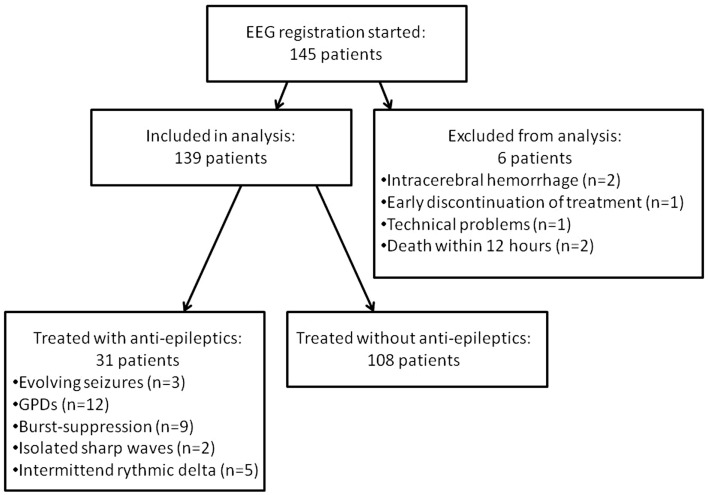
**Flowchart of included patients**. GPDs indicate generalized periodic discharges. Burst-suppression patterns consisted of bursts resembling epileptiform discharges of 1 up to 5 s.

As of March 31, 2013, 139 patients had been included (108 in Medisch Spectrum Twente and 31 in Rijnstate Hospital, Figure 1). Baseline characteristics are presented in Table 1. Blinded EEG evaluation at 24 h could be performed in 121 patients. Analysis at 24 h was not possible in 18 patients due to artifacts in the automatically selected 5 min epochs.

**Table 1:** **Baseline characteristics of patients treated with and without anti-epileptic drugs**.

	Treatment with anti-epileptic drugs		*P*-value
	Yes (*n* = 31)	No (*n* = 108)	
Age (mean years ± SD)	64 ± 11	65 ± 12	0.6
OHCA	29	95	0.4
Presumed cause of cardiac arrest			0.7
Cardiac	20	82	
Other	5	11	
Unknown	6	15	
Initial rhythm			0.7
VF	21	76	
Asystole	6	17	
Bradycardia	2	4	
Unknown	2	11	
Propofol treatment	28	101	0.7
Propofol dosage (mg/kg/h, mean ± SD)	3.0 ± 0.7	2.8 ± 1.1	
Midazolam treatment	9	36	0.9
Midazolam dosage (μg/kg/h, mean ± SD)	211 ± 271	309 ± 252	
Fentanyl treatment	17	53	0.4
Fentanyl dosage (μg/kg/h, mean ± SD)	1.6 ± 0.7	1.8 ± 0.8	
Remifentanil treatment	9	33	0.8
Remifentanil dosage (μg/kg/h, mean ± SD)	4.7 ± 2.3	4.2 ± 0.7	
Morphine treatment	3	23	0.4
Morphine dosage (μg/kg/h, mean ± SD)	331 ± 148	309 ± 119	

*SD indicates standard deviation; OHCA, out of hospital cardiac arrest; VF, ventricular fibrillation; dosage, maximum dosage within the first 24 h*.

Thirty-one patients (22%) were treated with anti-epileptic drugs. This treatment was initiated at a median of 47 h after cardiac arrest (interquartile range 36–76). Drugs and dosages were phenytoin initial dosage 1000–1500 mg followed by 200–300 mg daily in two doses, levetiracetam 1000–1500 mg daily in two doses, valproate initial dosage 1000–1800 mg followed by 1000–1500 mg daily in two doses, clonazepam single or repeated bolus of 1 mg, Propofol 200–400 mg/h, or Midazolam 8–10 mg/h. Two patients were treated with 1, 9 with 2, 13 with 3, 5 with 4, 1 with 5, and 1 with 6 different anti-epileptic drugs. If additional anti-epileptic drugs were added, previously given medication had not sufficiently improved epileptic EEG patterns. Treatment with conventional anti-epileptic drugs continued after discharge from the ICU in surviving patients. Barbiturates were not used.

**Figure 2:**
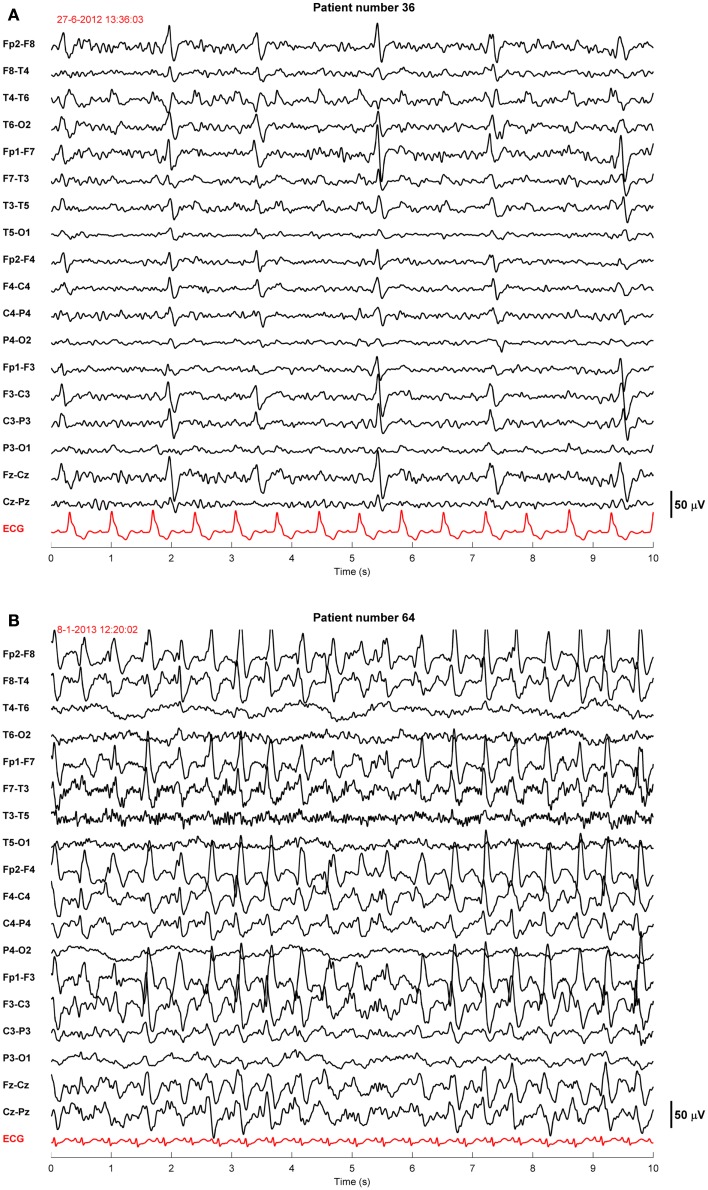
**Examples of EEGs of two comatose patients after cardiac arrest showing generalized periodic discharges**. These patients were normothermic and sedated with propofol 1–2.5 mg/kg/h. The EEG epochs were recorded 46 h **(A)** or 68 h **(B)** after cardiac arrest. Filter settings, 0.5–30 Hz. These patients had a poor outcome.

**Figure 3:**
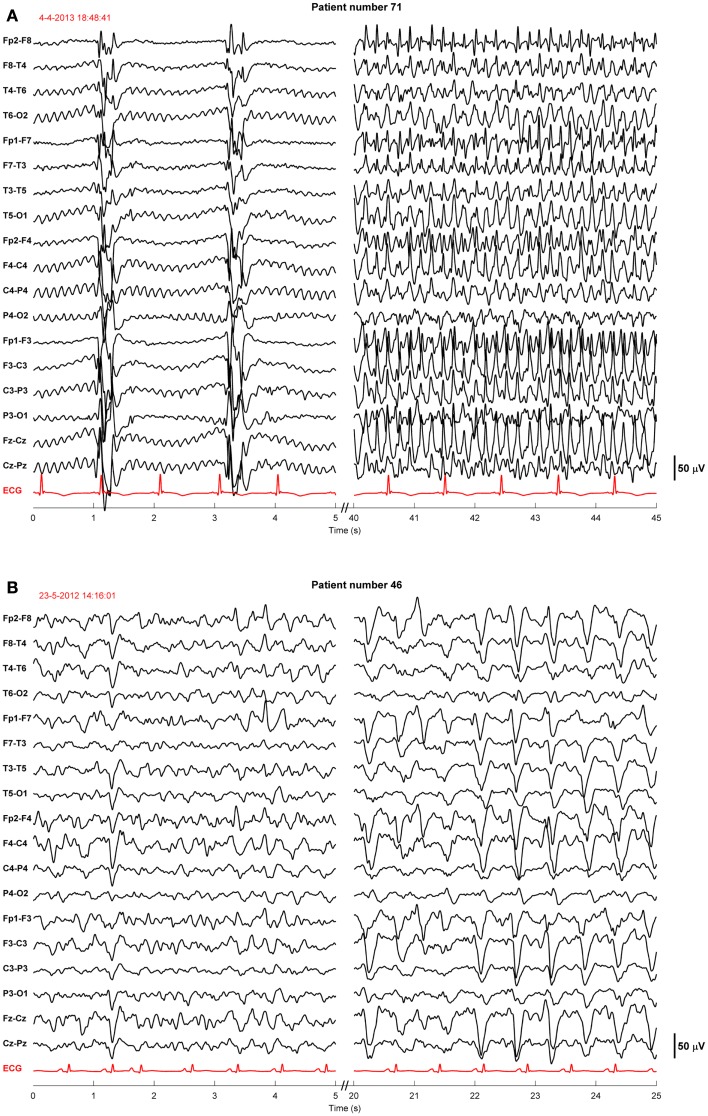
**Examples of EEGs of two comatose patients after cardiac arrest showing evolving seizures**. These patients were sedated with propofol 1–2.5 mg/kg/h. The EEG epochs were recorded 19 h after cardiac arrest, during therapeutic hypothermia (33°C) **(A)**, or 78 h after cardiac arrest, after restoration of normothermia **(B)**. Filter settings, 0.5–30 Hz. These patients had a poor outcome.

**Figure 4:**
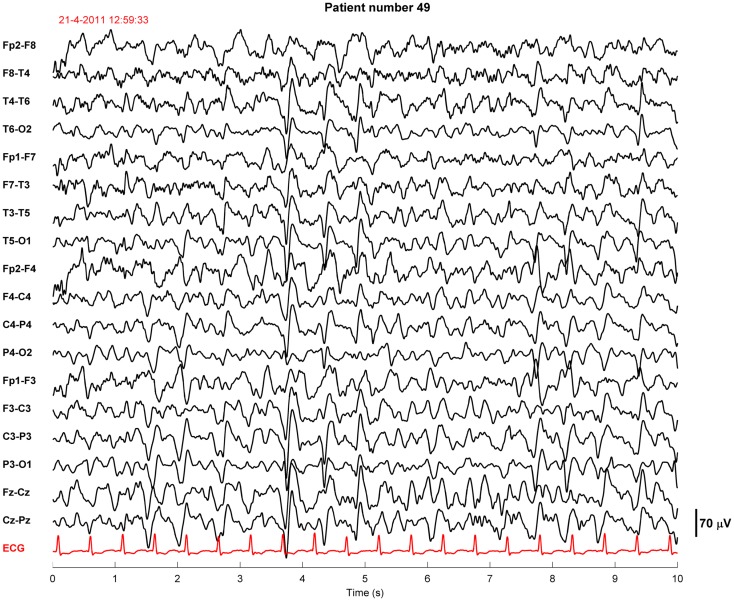
**Example of an EEG fragment of a comatose patient after cardiac arrest showing generalized periodic discharges intermixed with non-rhythmic activity**. This patient was sedated with propofol 2.8 mg/kg/h. The EEG epoch was recorded approximately 40 h after cardiac arrest, after restoration of normothermia. Filter settings, 0.5–30 Hz. This patient had a good outcome.

Twenty-four of the treated patients fulfilled criteria for electroencephalographic status epilepticus: 3 had evolving seizures, 12 GPDs, and 9 burst-suppression with bursts resembling epileptiform discharges during more than 30 min. Electroencephalographic status epilepticus, including evolving seizures, GPDs, and burst-suppression, started at a median of 28 h after cardiac arrest (interquartile range 23–37). Examples are shown in Figures 2 and 3. All 24 identified patients with a status epilepticus were treated with additional anesthetics: 23 with propofol, 1 with midazolam, and 11 with a combination of propofol and midazolam. In these patients, treatment improved the EEG patterns up to temporary suppression of epileptiform activity, but never extending a period of 6 h. Medication induced burst-suppression was never achieved. All but one patient with status epilepticus, treated with anti-epileptic drugs, had a poor outcome and died (Table 2). The only patient with a status epilepticus and a good outcome (CPC score of 1 at 6 months) had GPDs intermixed with apparently physiological activity. This patient was treated with valproate, 1000 mg/day, and propofol, 2.8 mg/kg/h (Figure 4).

**Table 2:** **Proportions of patients with improved EEG or poor outcome after treatment with (combinations of) anti-epileptic drugs, according to the EEG pattern at the initiation of treatment**.

EEG pattern at initiation of treatment (*n*)	Improved EEG *n* (%)	Poor outcome *n* (%)
Evolving seizures (3)	3 (100)	3 (100)
GPD (12)	9 (75)	11 (92)^b^
Burst-suppression^a^ (9)	3 (33)	9 (100)
Isolated sharp waves (2)	2 (100)	0
Intermittent rhythmic delta (5)	5 (100)	0

*Two patients were treated with 1, 9 with 2, 13 with 3, 5 with 4, 1 with 5, and 1 with 6 different anti-epileptic drugs*.

*^a^Burst-suppression patterns consisted of bursts resembling epileptiform discharges of 1 up to 5 s. Complete suppression of epileptiform patterns never lasted longer than 6 h; EEG, electroencephalography; n.a., not accessible*.

*^b^In the only patient with GPDs and a good outcome, GPDs were intermixed with apparently physiological activity*.

Seven of the treated patients had paroxysmal epileptiform activity, but not electroencephalographic status epilepticus: five had short (3–10 s) episodes of rhythmic delta activity, and three had isolated sharp waves, both superimposed on diffusely slowed, continuous patterns. These patients all had a good outcome: three had a CPC score of 1 and 4 of 2 at 6 months (Table 2).

Patients treated with and without anti-epileptic drugs are compared with regard to the risk of poor outcome (Table 3). There were no statistically significant differences in the subgroup with status epilepticus at 24 h (evolving seizures or GPDs). Otherwise, in patients with diffusely slowed or normal EEG patterns at 24 h after cardiac arrest, the proportion of patients with a poor outcome was lower after treatment with anti-epileptic drugs.

**Table 3:** **Proportions of patients with poor outcome treated with or without anti-epileptic drugs according to EEG pattern at 24 h after cardiac arrest**.

EEG pattern at 24 h	Poor outcome with AED *n*/*N* (%)	Poor outcome without AED *n*/*N* (%)	OR (95% CI)
Iso-electric or low voltage (*n* = 12)	5/5 (100)	7/7 (100)	n.a.
Evolving seizures, GPD, or burst-suppression (*n* = 46)	14/17 (82)	23/29 (79)	1.1 (0.4–3.1)
Continuously slowed (*n* = 61)	0/5 (0)	7/54 (13)	0.9 (0.8–1.0)

*AED indicates anti-epileptic drugs; OR, odds ratio of poor outcome of patients treated with as compared to patients treated without AED; 95% CI, 95% confidence interval*.

## Discussion

Retrospective analysis of treatment with anti-epileptic drugs in our prospectively collected cohort of comatose patients after cardiac arrest demonstrates that unstandardized, moderate treatment with conventional anti-epileptic drugs, not leading to complete suppression of epileptiform patterns for longer than 6 h, is common practice. There was no evidence for a beneficial effect of such treatment on outcome of patients with electroencephalographic status epilepticus.

Many of our patients who were treated with anti-epileptic drugs fulfilled the criteria for status epilepticus by semiology, EEG appearance, and duration. Still, all were treated only moderately intensive. In our cohort, moderate treatment indicated that most patients received solely conventional anti-epileptic drugs in standard doses. If propofol or midazolam was used, dosages were never sufficient to suppress epileptiform activity for more than 6 h. Barbiturates were not used and medication induced burst-suppression was never achieved. A more aggressive treatment of status epilepticus improves outcome if directed at complete suppression of electroencephalographic epileptiform discharges during at least 24 h (21). Whether this holds for status epilepticus in post-anoxic encephalopathy after cardiac arrest is unknown. Nevertheless, the moderate character of treatment in our cohort seems to be common practice and representative for the worldwide ambivalence toward treatment of electroencephalographic status epilepticus in this patient group (18, 20). This moderation reflects the uncertainty with regard to the use of any treatment.

Apart from the intensity of treatment, the onset of treatment probably plays an important role. With continuous EEG monitoring starting 12 h after cardiac arrest, we and others found that in approximately one-quarter of patients with electroencephalographic status epilepticus, the epileptiform patterns started before 24 h after cardiac arrest (4, 5, 22). In previous studies, EEG monitoring only started at a median of 2–3 days after cardiac arrest, indicating that diagnosis and subsequent treatment of electroencephalographic status epilepticus started thereafter at its earliest (6, 10, 12). In our current study, the median time to onset of electroencephalographic status epilepticus was 28 h, whereas the median time to treatment was 47 h. Mechanisms such as excessive glutamate release are known to worsen brain damage in ongoing status epilepticus within 20–40 min (23). Also, prolonged duration of status epilepticus reduces the effect of treatment, e.g., due to receptor trafficking (24). Thus, the initiation of treatment many hours after the onset of electroencephalographic status epilepticus may be too late to prevent irreversible damage.

Previous studies have focused on electroencephalographic status epilepticus as a predictor of poor outcome after cardiac arrest and the identification of patients in whom treatment of status epilepticus might be beneficial. These have shown that sporadic patients with post-anoxic encephalopathy after cardiac arrest and electroencephalographic status epilepticus may survive (4, 12, 13, 25). Identified possible determinants of a favorable outcome include a continuous background pattern (25), preserved brainstem reactions, and EEG reactivity (6). However, even in survivors, it remained unclear whether or not (aggressive) treatment had improved outcome, since electroencephalographic status epilepticus after cardiac arrest is often spontaneously transient (6).

The evidence of a possible beneficial effect of anti-epileptic drugs on outcome of patients with relatively favorable EEG patterns is weak, since groups are small and bias by indication, with selective treatment of patients with a relatively good prognosis, cannot be excluded. The only neuroprotective treatment of proven benefit so far in comatose patients after cardiac arrest is mild therapeutic hypothermia (1). A randomized controlled trial on the effect of prophylactic treatment with anti-epileptic drugs is ongoing^1^.

This study has limitations. First, although data on patient outcome and EEG patterns were pre-defined and collected prospectively, data on the use of anti-epileptic drugs were retrieved retrospectively, implying possible observation or selection bias. Second, since evidence of effect for treatment with anti-epileptic drugs is lacking, there was no treatment protocol. Therefore, both the nature and the intensity of treatment differed among physicians. However, treatment never reached an intensity to induce burst-suppression EEG and barbiturates were not used. Third, in patients who were *not* treated with anti-epileptic drugs, the presence of epileptiform EEG patterns was assessed by random checks at 24 h after cardiac arrest. This indicates that the incidence of epileptiform patterns was possibly underestimated. For this reason, comparisons between patients treated with and without anti-epileptic drugs presented in Table 3 should be interpreted with caution. Fourth, although the Glasgow Coma Scale score was measured daily, information on other clinical parameters had not been collected prospectively, and retrospective collection appeared unreliable. Therefore, the proportion of patients with clinically overt myoclonic status epilepticus was unclear. However, in patients after cardiac arrest, for both electroencephalographic seizures and clinical myoclonia it is not clear whether these represent “true” seizures, with a possibility to return to physiological activity, or an expression of severe (irreversible) damage (26). For most neurologists, the threshold to treat patients with overt myoclonia is lower than for patients with non-convulsive electroencephalographic seizures. However, irreversible damage is probably even more likely in patients with myoclonia, since the risk of poor outcome is larger (6) and neuronal necrosis is more common (26). Fifth, we selected patients based on treatment with specific anti-epileptic drugs and only identified continuously infused propofol or midazolam as a treatment against electroencephalographic seizures, if dosages increased simultaneously with the initiation of treatment with anti-epileptic drugs. We cannot exclude that in some other patients electroencephalographic seizures were treated solely with propofol or midazolam.

## Conclusion

In comatose patients after cardiac arrest with electroencephalographic status epilepticus, unstandardized treatment with conventional anti-epileptic drugs in standard doses, that only suppresses pathological EEG patterns temporarily (<6 h), is common practice. Although widely used, such treatment does probably not improve patients’ outcome. A randomized controlled trial to estimate the effect of early and aggressive treatment, directed at complete suppression of epileptiform activity during at least 24 h, is needed and in preparation (unique identifier NCT02056236)^2^.

## Author Contributions

Jeannette Hofmeijer, Marleen C. Tjepkema-Cloostermans, Michel J. A. M. van Putten: study design and conceptualization, data interpretation and analysis, and writing or revising the manuscript. Michiel J. Blans, Albertus Beishuizen: data interpretation and analysis, and writing or revising the manuscript.

## Conflict of Interest Statement

The authors declare that the research was conducted in the absence of any commercial or financial relationships that could be construed as a potential conflict of interest.

## References

[1] Bernard SA Gray TW Buist MD Jones BM Silvester W Gutteridge G Treatment of comatose survivors of out-of-hospital cardiac arrest with induced hypothermia. N Engl J Med (2002) 346:557–63 10.1056/NEJMoa003289 11856794

[2] Krumholz A Stern BJ Weiss HD Outcome from coma after cardiopulmonary resuscitation: relation to seizures and myoclonus. Neurology (1988) 38:401–5 10.1212/WNL.38.3.401 3347343

[3] Zandbergen EG de Haan RJ Stoutenbeek CP Koelman JH Hijdra A Systematic review of early prediction of poor outcome in anoxic-ischaemic coma. Lancet (1998) 352:1808–12 10.1016/S0140-6736(98)04076-8 9851380

[4] Cloostermans MC van Meulen FB Eertman CJ Hom HW van Putten MJ Continuous electroencephalography monitoring for early prediction of neurological outcome in postanoxic patients after cardiac arrest: a prospective cohort study. Crit Care Med (2012) 40:2867–75 10.1097/CCM.0b013e31825b94f0 22824933

[5] Rittenberger JC Popescu A Brenner RP Guyette FX Callaway CW Frequency and timing of nonconvulsive status epilepticus in comatose post-cardiac arrest subjects treated with hypothermia. Neurocrit Care (2012) 16:114–22 10.1007/s12028-011-9565-0 21638118 PMC3188346

[6] Rossetti AO Oddo M Liaudet L Kaplan PW Predictors of awakening from postanoxic status epilepticus after therapeutic hypothermia. Neurology (2009) 72:744–9 10.1212/01.wnl.0000343006.60851.62 19237704

[7] Zandbergen EG Hijdra A Koelman JH Hart AA Vos PE Verbeek MM Prediction of poor outcome within the first 3 days of postanoxic coma. Neurology (2006) 66:62–8 10.1212/01.wnl.0000191308.22233.88 16401847

[8] Celesia GG Grigg MM Ross E Generalized status myoclonicus in acute anoxic and toxic-metabolic encephalopathies. Arch Neurol (1988) 45:781–4 10.1001/archneur.1988.00520310099023 3390032

[9] Hui AC Cheng C Lam A Mok V Joynt GM Prognosis following postanoxic myoclonus status epilepticus. Eur Neurol (2005) 54:10–3 10.1159/000086755 16015015

[10] Kaplan PW Morales Y Status epilepticus: an independent outcome predictor after cerebral anoxia. Neurology (2008) 70:1295–6 10.1212/01.wnl.0000312074.77793.a4 18391164

[11] Legriel S Bruneel F Sediri H Hilly J Abbosh N Lagarrigue MH Early EEG monitoring for detecting postanoxic status epilepticus during therapeutic hypothermia: a pilot study. Neurocrit Care (2009) 11:338–44 10.1007/s12028-009-9246-4 19588273

[12] Rossetti AO Logroscino G Liaudet L Ruffieux C Ribordy V Schaller MD Status epilepticus: an independent outcome predictor after cerebral anoxia. Neurology (2007) 69:255–60 10.1212/01.wnl.0000265819.36639.e0 17636063

[13] San-Juan OD Chiappa KH Costello DJ Cole AJ Periodic epileptiform discharges in hypoxic encephalopathy: BiPLEDs and GPEDs as a poor prognosis for survival. Seizure (2009) 18:365–8 10.1016/j.seizure.2009.01.003 19196524

[14] Brenner RP Is it status? Epilepsia (2002) 43(Suppl 3):103–13 10.1046/j.1528-1157.43.s.3.9.x 12060012

[15] Chong DJ Hirsch LJ Which EEG patterns warrant treatment in the critically ill? Reviewing the evidence for treatment of periodic epileptiform discharges and related patterns. J Clin Neurophysiol (2005) 22:79–91 10.1097/01.WNP.0000158699.78529.AF 15805807

[16] Hirsch LJ Atlas of EEG in Critical Care. Wiley Blackwell (2010). 10.1002/9780470746707

[17] Tjepkema-Cloostermans MC Hindriks R Hofmeijer J van Putten M Generalized periodic discharges after acute cerebral ischemia: reflection of selective synaptic failure? Clin Neurophysiol (2014) 125(2):255–62 10.1016/j.clinph.2013.08.005 24012049

[18] Abend NS Dlugos DJ Hahn CD Hirsch LJ Herman ST Use of EEG monitoring and management of non-convulsive seizures in critically ill patients: a survey of neurologists. Neurocrit Care (2010) 12:382–9 10.1007/s12028-010-9337-2 20198513 PMC2944658

[19] Kilbride RD Costello DJ Chiappa KH How seizure detection by continuous electroencephalographic monitoring affects the prescribing of antiepileptic medications. Arch Neurol (2009) 66:723–8 10.1001/archneurol.2009.100 19506131

[20] Bouwes A Kuiper MA Hijdra A Horn J Induced hypothermia and determination of neurological outcome after CPR in ICUs in the Netherlands: results of a survey. Resuscitation (2010) 81:393–7 10.1016/j.resuscitation.2009.12.032 20122776

[21] Shorvon S Super-refractory status epilepticus: an approach to therapy in this difficult clinical situation. Epilepsia (2011) 52(Suppl 8):53–6 10.1111/j.1528-1167.2011.03238.x 21967364

[22] Mani R Schmitt SE Mazer M Putt ME Gaieski DF The frequency and timing of epileptiform activity on continuous electroencephalogram in comatose post cardiac arrest syndrome patients treated with therapeutic hypothermia. Resuscitation (2012) 83:840–7 10.1016/j.resuscitation.2012.02.015 22366352 PMC8851397

[23] Fujikawa DG Prolonged seizures and cellular injury: understanding the connection. Epilepsy Behav (2005) 7(Suppl 3):S3–11 10.1016/j.yebeh.2005.08.003 16278099

[24] Naylor DE Liu H Wasterlain CG Trafficking of GABA(A) receptors, loss of inhibition, and a mechanism for pharmacoresistance in status epilepticus. J Neurosci (2005) 25:7724–33 10.1523/JNEUROSCI.4944-04.2005 16120773 PMC6725248

[25] Rundgren M Westhall E Cronberg T Rosen I Friberg H Continuous amplitude-integrated electroencephalogram predicts outcome in hypothermia-treated cardiac arrest patients. Crit Care Med (2010) 38:1838–44 10.1097/CCM.0b013e3181eaa1e7 20562694

[26] Young GB Gilbert JJ Zochodne DW The significance of myoclonic status epilepticus in postanoxic coma. Neurology (1990) 40:1843–8 10.1212/WNL.40.12.1843 2123307

